# Supplementary material for: Estimation of Genetic Parameters for Conformation Traits and Milk Production Traits in Chinese Holsteins
Source: Animals (Basel). 2022 Dec 27;13(1):100. doi: 10.3390/ani13010100 (PMC9817994; doi:10.3390/ani13010100)
Supplement: Supplementary file 1 [file animals-13-00100-s001.zip › Supplementary Files/Figure S1.pdf]

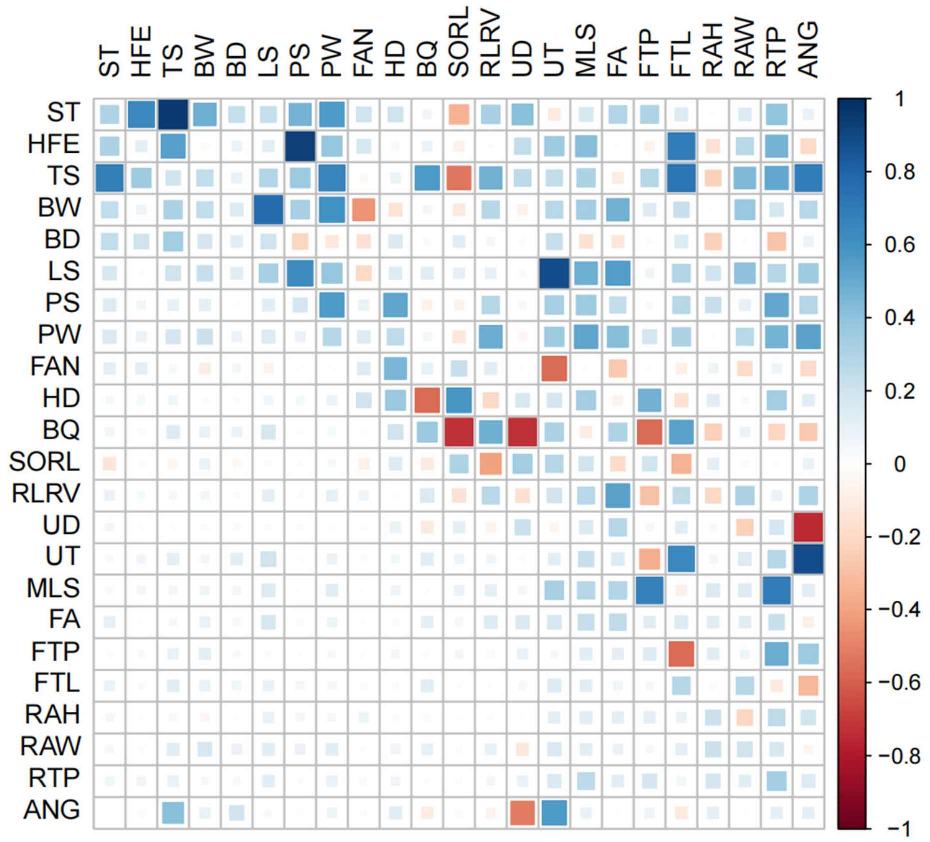

Figure S1. Genetic and phenotypic correlations among conformation traits. ST, stature; HFE, height at the front end; TS, trunk size; CW, chest width; BD, body depth; LS, loin strength; PS, pin setting; PW, pin width; FAN, foot angle; HD, heel depth; BQ, bone quality; SORL, set of rear legs; RLRV, rear leg-rear view; UD, udder depth; UT, udder texture; MSL, median sus-pensory; FA, fore attachment; FTP, fore teat placement; FTL, fore teat length; RAH, rear attachment height; RAW, rear attachment width; RTP, rear teat placement; ANG, angularity. Blue represents a positive correlation and red represents a negative correlation.
