# Supplementary material for: Estimation of Genetic Parameters for Conformation Traits and Milk Production Traits in Chinese Holsteins
Source: Animals (Basel). 2022 Dec 27;13(1):100. doi: 10.3390/ani13010100 (PMC9817994; doi:10.3390/ani13010100)
Supplement: Supplementary file 1 [file animals-13-00100-s001.zip › Supplementary Files/Table S1.pdf]

Table S1. Genetic and phenotypic correlations between conformation traits[1] in Chinese Holsteins.

Traits[2]	ST	HFE	TS	CW	BD	LS	PS	PW	FAN	HD	BQ	SORL	RLRV	UD	UT	MSL	FA	FTP	FTL	RAH	RAW	RTP	ANG
ST		0.64	**0.95**	0.48	0.23	0.23	0.46	0.57	0.20	0.20	0.06	-0.35	0.32	0.41	-0.12	0.17	0.29	0.30	0.15	-0.01	0.14	0.39	0.09
HFE	0.31		**0.54**	-0.03	0.08	0.11	**0.93**	0.38	0.17	-0.04	0.08	-0.08	0.01	0.24	0.35	0.42	0.01	-0.07	**0.69**	-0.16	0.27	0.46	-0.20
TS	**0.68**	0.35		0.24	0.09	0.29	0.36	**0.65**	-0.04	0.08	**0.56**	-0.53	0.47	0.26	0.24	0.31	-0.10	0.28	**0.72**	-0.23	0.44	**0.51**	**0.69**
CW	0.24	0.07	0.31		0.15	**0.76**	0.32	**0.60**	-0.45	-0.15	0.05	-0.12	0.28	-0.08	0.27	0.34	0.47	0.14	0.22	-0.01	0.37	0.17	0.27
BD	0.24	0.20	0.34	0.18		0.20	-0.22	-0.13	-0.16	0.16	0.03	0.12	0.05	0.02	0.23	-0.16	-0.13	-0.02	0.15	-0.24	0.01	-0.29	0.08
LS	0.17	0.01	0.20	0.22	0.13		**0.62**	0.38	-0.20	0.14	0.13	0.10	0.10	0.03	**0.88**	0.48	**0.55**	0.06	0.29	0.19	0.40	0.28	0.35
PS	0.14	0.05	0.10	0.11	-0.01	0.13		**0.56**	0.09	0.52	-0.08	-0.07	0.28	0.04	0.32	0.36	0.24	0.03	0.27	0.22	0.10	0.51	0.28
PW	0.16	0.08	0.18	0.21	0.08	0.15	0.08		0.14	0.25	0.05	-0.13	0.49	-0.05	0.35	**0.52**	0.42	0.18	0.31	-0.01	0.27	0.46	**0.53**
FAN	0.11	0.11	0.04	-0.10	0.05	-0.07	-0.01	-0.01		0.45	0.10	0.22	0.13	0.01	-0.57	0.01	-0.27	-0.01	-0.09	0.07	-0.19	0.07	-0.20
HD	0.04	0.04	0.06	0.02	0.02	0.06	0.04	0.08	0.20		-0.56	**0.58**	-0.21	0.17	0.18	0.34	-0.07	0.47	-0.16	0.13	0.03	0.34	0.12
BQ	0.05	-0.03	0.14	0.09	0.08	0.17	0.01	0.04	-0.02	0.20		-0.73	0.48	-0.73	0.31	-0.11	0.30	-0.56	0.53	-0.24	0.08	-0.22	-0.27
SORL	-0.14	0.02	-0.06	0.09	-0.04	0.02	0.01	0.04	-0.09	0.14	-0.12		-0.41	0.34	0.28	0.19	-0.19	0.19	-0.34	0.11	-0.03	-0.02	0.09
RLRV	0.08	0.04	0.04	0.05	0.02	0.11	-0.02	0.10	0.07	0.01	0.16	-0.16		-0.17	0.19	0.27	0.53	-0.30	0.25	-0.21	0.31	0.08	0.30
UD	0.05	0.01	0.01	0.02	0.03	0.02	0.02	-0.02	-0.02	0.09	-0.12	0.11	-0.07		-0.06	0.16	0.28	0.04	0.12	0.04	-0.24	0.18	-0.75
UT	0.03	0.06	0.11	0.05	0.12	0.20	-0.01	0.08	-0.03	0.05	0.13	0.07	0.06	0.02		0.22	0.14	-0.37	**0.64**	0.05	0.15	0.28	**0.88**
MSL	0.03	0.05	0.05	0.06	0.05	0.15	-0.01	0.12	-0.01	0.07	0.01	0.08	0.11	0.05	0.32		0.29	**0.67**	-0.09	0.16	0.15	**0.69**	0.13
FA	0.10	0.01	0.04	0.09	0.04	0.17	-0.03	0.07	0.01	0.02	0.11	-0.05	0.14	0.16	0.17	0.23		0.12	0.15	0.12	0.12	0.22	-0.09
FTP	0.02	0.03	0.09	0.11	0.03	0.02	0.01	0.04	0.00	0.05	-0.02	0.05	-0.03	0.09	0.04	0.13	0.11		-0.57	0.12	0.06	0.49	0.36
FTL	0.08	0.03	0.12	0.09	0.06	0.09	0.05	0.05	-0.03	0.02	0.13	0.00	0.01	0.04	0.15	0.12	0.06	0.04		-0.03	0.28	-0.12	-0.33
RAH	0.04	0.01	-0.04	-0.05	0.01	0.09	0.05	0.04	0.07	0.02	-0.03	-0.01	-0.02	0.01	0.11	0.13	0.11	0.11	0.08		-0.22	0.25	0.19
RAW	0.04	0.02	0.13	0.17	0.08	0.13	0.08	0.12	-0.02	0.07	0.10	0.02	0.10	-0.13	0.16	0.12	0.08	0.04	0.11	0.21		0.17	-0.06
RTP	0.06	0.06	0.09	0.04	0.03	0.12	0.02	0.09	0.03	0.06	0.02	0.02	0.05	0.09	0.16	0.26	0.12	0.18	0.05	0.18	0.12		0.15
ANG	0.00	-0.03	**0.41**	0.09	0.19	0.03	-0.01	0.07	0.02	0.13	-0.11	-0.03	-0.07	-0.52	**0.56**	0.09	-0.01	0.07	-0.12	0.11	-0.01	0.11	

[1] The genetic correlation (above diagonal), heritability (diagonal), and phenotypic correlation (below diagonal).

[2] ST, stature; HFE, height at the front end; TS, trunk size; CW, chest width; BD, body depth; LS, loin strength; PS, pin setting; PW, pin width; FAN, foot angle; HD, heel depth; BQ, bone quality; SORL, set of rear legs; RLRV, rear leg-rear view; UD, udder depth; UT, udder texture; MSL, median suspensory; FA, fore attachment; FTP, fore teat placement; FTL, fore teat length; RAH, rear attachment height; RAW, rear attachment width; RTP, rear teat placement; ANG, angularity.
